# Harnessing Phenotypic Plasticity to Improve Maize Yields

**DOI:** 10.3389/fpls.2018.01377

**Published:** 2018-09-19

**Authors:** Aaron Kusmec, Natalia de Leon, Patrick S. Schnable

**Affiliations:** ^1^Department of Agronomy, Iowa State University, Ames, IA, United States; ^2^Department of Agronomy, University of Wisconsin-Madison, Madison, WI, United States; ^3^Plant Sciences Institute, Iowa State University, Ames, IA, United States

**Keywords:** genotype–environment interactions, phenotypic plasticity, artificial selection, genetic architecture, maize

## Abstract

Plants can produce different phenotypes when exposed to different environments. Understanding the genetic basis of these plastic responses is crucial for crop breeding efforts. We discuss two recent studies that suggest that yield plasticity in maize has been under selection but is controlled by different genes than yield.

## Introduction

**FIGURE 1:**
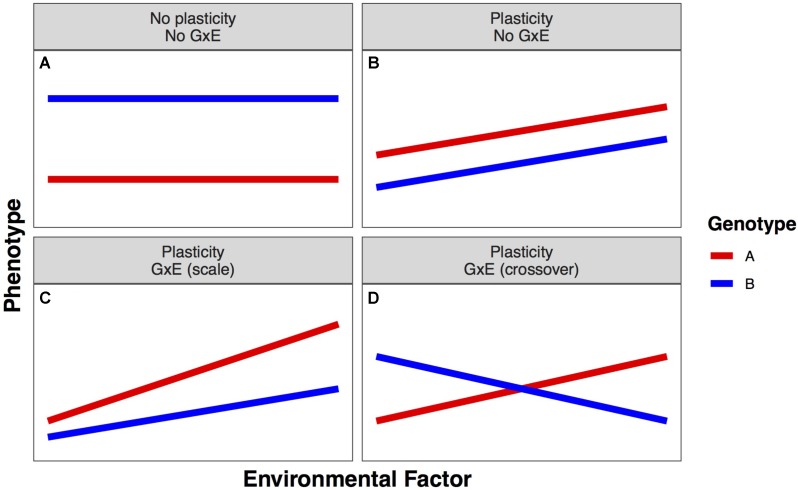
Example forms of linear plastic responses. **(A)** Neither genotype expresses different phenotypes in different environments. **(B)** Both genotypes exhibit the same plastic response, so there is no genotype–environment interaction (GxE). **(C)** Both genotypes exhibit different plastic responses that lead to a greater advantage of genotype A over genotype B as the environmental factor increases (scale GxE). **(D)** Both genotypes exhibit different plastic responses that cause the best genotype to change in different environments (crossover GxE).

Phenotypic plasticity is phenotypic variation that results from the complex relationships between an individual’s genotype (G) and the environment (E), including management decisions, in which it is grown. The ability of a single genotype to produce different phenotypes in different environments is termed phenotypic plasticity. The amount of phenotypic change across environments describes the degree of plasticity. When this change is far from zero, the phenotype is plastic; when this change is near zero, the phenotype is stable. Phenotypic plasticity is a characteristic of an individual genotype; when variation for plasticity exists within a population, it is termed genotype-by-environment interaction (GxE) (**Figure 1**; Bradshaw, 1965).

Phenotypic plasticity is often contrasted with a concept from developmental biology, termed canalization, although the two terms are not strictly opposites. Phenotypic variation arises from an organism’s genotype, its environment, its developmental trajectory, and interactions among these three factors. The more diverse the genotypes that are studied and the environments in which they are measured, the more phenotypic variation that is expected to be observed. If a single genotype is measured in many environments, while different developmental trajectories may be involved, the principal source of phenotypic variation is due to plastic responses (or lack thereof). Responses to this macroenvironmental variation are termed plasticity (Pigliucci, 2001). By contrast, if a single genotype is measured multiple times in those environments, a second source of phenotypic variation can be observed: developmental variation due to minor environmental fluctuations within a macroenvironment. The tendency of a genotype to produce the same phenotype regardless of this microenvironmental variation is termed canalization (Waddington, 1942).

The adaptive significance of phenotypic plasticity is situational (Bradshaw, 1965; Ghalambor et al., 2007). For example, cultivars (i.e., specific genotypes) that are stable with respect to common stresses in an environment are valuable for their predictable and consistent yields. However, such stability would be less valuable in an environment where stresses are well controlled and cultivars, via sufficiently plastic responses, could produce higher yields by taking advantage of favorable environmental conditions.

Consequently, accounting for plastic responses in applications such as plant breeding is critical because plants are sessile and must respond to the environments in which they are grown. Breeders have approached this challenge in two ways (Bernardo, 2010). First, breeders can reduce plasticity by selecting cultivars that produce stable yields across a range of relatively homogeneous environments. Second, breeders can exploit plasticity by selecting cultivars that produce high yields in some environments at the expense of lower yields in other environments. As breeders develop cultivars adapted to future environments, they are constrained by the impact of past selection decisions and the genetic relationships between average yield across environments and plasticity.

Information about the genetic basis of plasticity is required to reduce or exploit it. Numerous studies have explored the genetic architecture of phenotypic plasticity in natural and crop species (reviewed in Des Marais et al., 2013) and identified loci with significant QTL-by-environment interactions. These environment-specific QTL can be associated with continuously varying environmental factors in a *post hoc* manner (e.g., Millet et al., 2016) to overcome the biological limitations of treating environments as discrete entities. Another approach is to use joint regression analysis (Yates and Cochran, 1938; Finlay and Wilkinson, 1963) or factorial regression (van Eeuwijk, 1995) to quantify plastic responses with respect to environmental gradients (e.g., Kraakman et al., 2004; Emebiri and Moody, 2006; Lacaze et al., 2009).

These and other studies have identified QTLs that demonstrate different effects in different environments (allelic sensitivity model) and QTLs that only have effects in certain environments (gene regulation model) (Via et al., 1995). Controversy arose in the 1980s and 1990s over which of these two models was “the” genetic model for plasticity. Now it is accepted that it is likely that both of these models contribute to the genetic basis for phenotypic plasticity, but to different extents depending on the organism, phenotype, and assayed environments (Pigliucci, 2001). Many of these earlier studies used small populations genotyped with few genetic markers and that were measured for only a few phenotypes in a small number of environments. Recent advances in genotyping and phenotyping technologies allow the dissection of the genetic architecture of phenotypic plasticity at ever-finer scales, especially in crop species, which are of both scientific and socioeconomic interest.

Maize is an ideal model and crop species in which to study phenotypic plasticity. It was domesticated in the lowlands of southwestern Mexico and subsequently adapted to highland and temperate environments (Piperno et al., 2009). Further adaptation has resulted from intensive public and private breeding programs over the last 100 years (Duvick, 2005) that has led to cultivars with varying degrees of stability or plasticity (Simmonds, 1981; Lobell et al., 2014). Additionally, large, diverse inbred and hybrid populations exist that can be replicated across environments to capture GxE variation. Two recent papers exploited this demographic history to explore the impacts of selection and genetic architecture on phenotypic plasticity in maize.

## Selection On Phenotypic Plasticity In Maize And Its Genetic Architecture

Gage et al. (2017) explored the impact of past selection decisions by identifying single nucleotide polymorphisms (SNPs) that exhibited evidence of selection (high *F_ST_* and low nucleotide diversity) between 30 temperate and 30 tropical inbred maize lines along with a control group of SNPs that exhibited no such evidence. These SNPs were used to test the amount of phenotypic variation among 858 temperate maize hybrids that could be explained by SNP-environment interactions within each group. They found that high *F_ST_* SNPs explained an average of 8.1% of the GxE variance for yield compared to 18.7% for low *F_ST_* SNPs. High *F_ST_* SNPs also explained less genetic variance for yield than low *F_ST_* SNPs. This suggests that selection, especially in genomic regions showing high differentiation between temperate and tropical germplasm, has increased yields in temperate environments at the expense of the amount of GxE variation associated with yield that is explained by those SNPs. This reduction in GxE variance could be the result of increased uniformity in either the yield plasticity or yield stability of modern temperate maize germplasm. Because breeders have heavily selected for yield stability in modern temperate maize, Gage et al. (2017) interpret the reduction in GxE variance as evidence that temperate maize yields have become more stable. While stable, predictable yields are beneficial, Gage et al. (2017) note that the loss of allelic diversity associated with yield plasticity may constrain adaptation to future environments affected by global climate change. Because these SNPs capture differentiation within genomic regions, it is possible that the reduction in both genetic and GxE variance explained by these SNPs is due to selection for genes that affect both yield and yield stability simultaneously or at multiple genes that affect yield and yield stability separately.

To address this question, Kusmec et al. (2017) explored the relationships between the genetic architectures of average traits across environments and plasticity for 23 phenotypes in a panel of ∼5,000 maize recombinant inbred lines (RILs). These phenotypes encompassed morphological (e.g., plant height), developmental (e.g., flowering time), and fitness (e.g., kernel row number and other yield components) traits. Through the combination of stability analysis and GWAS, they concluded that average traits and plasticity were controlled by structurally and functionally distinct sets of genes. This suggests the possibility of exploiting GxE by optimizing plasticity and increasing yields via independent selection on the different sets of genes in genomic regions where such genes are not genetically linked.

## A Genetic Model For Phenotypic Plasticity

If loci for yield and yield stability are genetically linked, selection for yield would be expected to affect allele frequencies at loci for yield stability (i.e., the relative absence of plasticity) and vice versa. Because breeders have selected for both yield and yield stability, the simultaneous reduction in genetic and GxE variances explained by putatively selected genomic regions suggests that these regions contain genetically linked genes for yield and yield stability where the favorable alleles for both traits are in coupling linkage. Simultaneous selection for yield and yield stability by breeders has been remarkably successful; for example, modern commercial hybrids have improved drought tolerance (i.e., increased stability) while also increasing yields in well-watered and drought conditions (Cooper et al., 2014). However, because yield is a highly polygenic trait, we also expect to find instances of yield and yield stability-associated loci that are unlinked or in repulsion linkage. Large-scale experiments, such as the Genomes to Fields (G2F) Initiative^1^, will be required to test this hypothesis.

Additionally, yield integrates plastic responses to multiple abiotic and biotic stresses; the genes that mediate each plastic response may or may not be linked to yield-related genes and/or each other, increasing the complexity of breeding decisions when selecting for adaptive loci for particular combinations of environmental conditions. This view of yield as a function-valued trait of multiple environmental factors has a rich theoretical background (Stinchcombe et al., 2012), but many open questions remain such as which environmental factors are important, when are they important, to what degree do they interact, and what is the form of the response (i.e., linear or non-linear). Recent work in maize and sorghum offer different answers to these questions (Millet et al., 2016; Li et al., 2018; Messina et al., 2018).

Identifying such loci will allow for the identification and selection of desirable recombinants in elite breeding germplasm. However, if the findings of Gage et al. (2017) can be generalized to regions that have been selected following temperate adaptation, the introduction of alleles that confer plasticity or stability from exotic germplasm will be necessary to maximize future adaptation of maize hybrids. Useful allelic variation can be mined and introduced from diverse inbred lines and numerous landraces that are highly adapted to local environments throughout maize’s adapted range (Romero Navarro et al., 2017). Introgression of such non-elite material often decreases yield. This challenge can be mitigated by initiating pre-breeding programs (Gorjanc et al., 2016) to recombine high-yielding haplotypes with adaptive variation, or by using technologies such as genome editing to introduce adaptive variation directly (Jenko et al., 2015).

## Conclusion

Further experiments to identify adaptive variation in maize paired with emerging technologies present an opportunity to improve yields not only by increasing average yield but also by maintaining and manipulating genetic variation that helps individuals maximize productivity across different environments. New cultivars can then be tailored to expected future conditions and agronomic practices in their target performance environments. Such an approach could not only create higher-yielding cultivars that exploit favorable conditions but also higher-yielding, stable cultivars that provide predictable yields in challenging environments where subsistence farming is often found.

## Author Contributions

All authors contributed to the writing and editing of the manuscript.

## Conflict of Interest Statement

The authors declare that the research was conducted in the absence of any commercial or financial relationships that could be construed as a potential conflict of interest.

## Footnotes

1 http://www.genomes2fields.org/
